# The Exceptional Solubility of Cyclic Trimetaphosphate in the Presence of Mg^2+^ and Ca^2+^

**DOI:** 10.3390/life16010184

**Published:** 2026-01-22

**Authors:** Megan G. Bachant, Ulrich F. Müller

**Affiliations:** Department of Chemistry & Biochemistry, University of California, La Jolla, CA 92093, USA; mbachant@ucsd.edu

**Keywords:** polyphosphate, origin of life, solubility

## Abstract

Studying the origin of life requires identifying chemical and physical processes that could have supported early self-replicating and evolving molecular systems. Besides the requirement of information storage and transfer, an essential aspect is an energy source that could have thermodynamically driven the formation and replication of these molecular assemblies. Chemical energy sources such as cyclic trimetaphosphate are attractive because they could drive replication with relatively simple catalysts. Here, we focus on cyclic trimetaphosphate (cTmp), and compare its solubility in water to linear triphosphate, pyrophosphate, and phosphite when Mg^2+^ or Ca^2+^ are present. These solubilities are important for facilitating the reactions under prebiotically plausible conditions. The results showed that cTmp was soluble even at molar concentrations of Mg^2+^ and little precipitation with 200 mM Ca^2+^. In contrast, pyrophosphate and linear triphosphate precipitated efficiently even at low divalent metal ion concentrations. The precipitation of phosphate was pH-dependent, showing similar precipitation with Mg^2+^ and Ca^2+^ at a prebiotically plausible pH of 6.5. Phosphite was soluble at high Mg^2+^ concentrations but started precipitating with increasing Ca^2+^ concentration. At conditions that model Archaean seawater, cTmp was the most soluble of these compounds. Together, this experimental overview may help to identify promising conditions for lab-based investigations of phosphate-based energy metabolisms in early life forms.

## 1. Introduction

Studies on the origin of life focus on objects that existed on Earth more than 3.5 billion years ago [1,2]—but without direct evidence of the molecular makeup of these objects. Microfossils in sediments of that age do not reveal whether they contained peptides or nucleic acids or even what sequence or chemical modifications those macromolecules may have had. Therefore, scientists are focusing on clues from today’s biology, on constraints from the geochemical environment, and on chemical and physical principles that govern self-replicating and molecular systems in general. A hard constraint on any self-replicating system is the second law of thermodynamics [3,4]: for any change over time, entropy must be generated, and if a molecular system is locally increased in structure (i.e., locally decreases entropy), then it requires a thermodynamic driving force from outside of this molecular system [5]. Several energy sources were available to early life forms. Sunlight provides the energy source for today’s oxygenic and anoxygenic photosynthesis [6,7], proton gradients across membranes are used in today’s biology to drive ATP synthesis [8,9], temperature gradients and wet–dry cycles are able to drive condensation reactions [10,11] and the spatial separation of molecules [12], and chemical disequilibria in the environment including from sulfide, ammonia, and cyanate serve as energy sources for chemoautotrophs [13,14,15]. Here, we focus on polyphosphates as energy sources because polyphosphates are prebiotically plausible [16,17,18,19] and can be used by catalytic RNAs (ribozymes) as energy sources in metabolically coupled reactions [20,21].

Cyclic trimetaphosphate (cTmp) is the most active polyphosphorylation reagent of the polyphosphates [22]. It can be generated in a prebiotic environment in several different processes, including high-energy events such as volcanism or lightning, redox reactions involving phosphite [insert the recent https://doi.org/10.1126/sciadv.adz2567] [16,17,23,24,25] and the condensation of pyrophosphate with diamidophosphate [26]. Cyclic Tmp can also be formed under mildly acidic conditions from longer polyphosphates, especially in the presence of Mg^2+^ [27]. Once formed, cTmp is stable for years in aqueous solution near neutral pH [28,29]; this kinetic stability is ideal for an energy metabolite. Cyclic Tmp can react with nucleoside 5′-hydroxyl groups to form nucleoside 5′-triphosphates (NTPs) [30,31]. The uncatalyzed rate at neutral pH is slow and can be accelerated with Mg^2+^ or Ni^2+^ ions [31,32] and further by ribozymes [20,21]. Therefore, their prebiotic plausibility, kinetic stability, useability by catalytic RNAs, and direct conversion to biology’s NTPs make cTmp a plausible energy currency for early RNA-dominated life forms.

The potential utility of cTmp as early energy source [33] would have been supported by its solubility in aqueous solution. While condensed phosphates are soluble with divalent cations if their molar concentration is in excess of the divalent cation [33], a prebiotic scenario would likely have contained lower condensed phosphate concentrations than divalent cations, under which conditions most condensed phosphates precipitate [34]. However, there is one outstanding exception: cyclic trimetaphosphate (cTmp) is soluble at several 100 mM concentrations, even with a large excess of Mg^2+^ [20]. Since this observation does not seem to be well known in the literature (including current Google AI searches, which describe cTmp salts with Mg^2+^ as highly insoluble at the time of writing), we dedicated this study to clarifying this unique behavior of cTmp.

**Figure 1:**
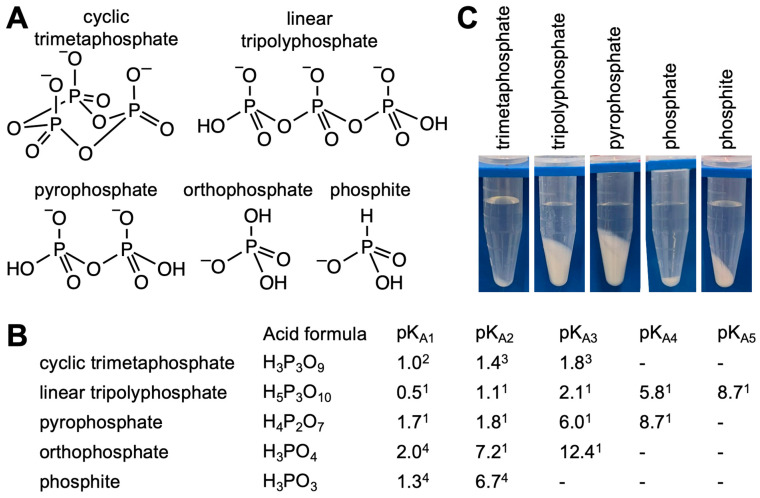
Phosphorus compounds and their precipitation investigated in this study. (**A**) The chemical structure of cyclic trimetaphosphate (cTm p), linear tripolyphosphate, pyrophosphate, orthophosphate, and phosphite. The protonation status corresponds to a pH of ~6 to illustrate the difference between protonation events in the strong acidic range (e.g., all three pK_A_s of cTmp) or in the strong alkaline range (e.g., pK_A3_ of orthophosphate). (**B**) pK_A_ values for the compounds shown in (**A**). The values are from ^1^ Thilo (1965) https://doi.org/10.1002/ange.19650772303 [27], ^2^ Davies & Monk (1949) https://doi.org/10.1039/JR9490000413 [29], ^3^ Figure S1, and ^4^ Larson and Pippin (1989) https://doi.org/10.1016/S0277-5387(00)80751-2 [35]). (**C**) Examples of experimental observations during the precipitation assay. The shown pellets resulted from the 100 mM phosphorus compound with 200 mM Ca^2+^, without pH buffering. The images show the 1 mL volume of the mixture in 1.5 mL centrifugation tubes after incubation for 20 h and centrifugation.

Here, we describe experimental results comparing the solubility of cTmp, linear tripolyphosphate, pyrophosphate, orthophosphate, and phosphite (Figure 1A) in the presence of Mg^2+^ and Ca^2+^ cations. The focus of this study is not to obtain thermodynamic constants but to cover a range of concentrations where precipitation is expected for the five phosphorus compounds and divalent cations and relate the results to prebiotically plausible conditions. These results should be useful for setting up experimental model systems for the involvement of condensed phosphates in the early stages of life.

## 2. Materials and Methods

### 2.1. Materials and Stock Solutions

All phosphorus compounds were used as sodium salts, and both divalent cation salts were used as chlorides. Cyclic trimetaphosphate was the trisodium salt from Aldrich (cat # T5508, Sigma-Aldrich, St. Louis, MO, USA), tripolyphosphate was the pentasodium salt from Acros Organics (cat # 393961000, Waltham, MA, USA), pyrophosphate was the tetrasodium salt from Alfa Aesar (cat # 33385, Waltham, MA, USA), the phosphate disodium salt was from Fisher (cat # S373, Fisher Scientific, Waltham, MA, USA), the phosphate monosodium salt was from EMD (cat # SX0710, Millipore, Burlington, MA, USA), and phosphite was the disodium salt from Acros Organics (cat # 428502500). Magnesium chloride (cat # M33) and calcium chloride (cat # C79) were from Fisher Scientific. All water was purified in a Millipore system. Stock solutions of the phosphorus species in water were prepared to a ‘round number’ close to the highest concentration that formed a clear solution. This was 500 mM for cyclic trimetaphosphate, 250 mM for linear tripolyphosphate, 175 mM for pyrophosphate, 500 mM for phosphate, and 500 mM for phosphite. The stock solution for phosphate at different pH values was prepared by mixing 500 mM Na_2_HPO_4_ with 500 mM NaH_2_PO_4_ in a 1:1 ratio for pH 7 and in a 9:1 ratio for pH 9.

### 2.2. Precipitation Assay

Each assay was prepared in a 1 mL volume in 1.5 mL centrifuge tubes after the empty tubes were weighed on a fine balance (Mettler Toledo AB54-S/FACT, Mettler-Toledo, Columbus, OH, USA). The most concentrated stock solution was entered first, followed by the addition of water, short vortexing, then the addition of the more dilute stock solution and vortexing for at least 10 s. For example, for the precipitation of 100 mM tetrasodium pyrophosphate (Figure 1C), with 500 mM MgCl_2_, 250 μL 2 M MgCl_2_ was pipetted into the tube, followed by 179 μL of water, vortexing, then adding 571 μL of 175 mM tetrasodium pyrophosphate and renewed vortexing for 10 s. In several cases, a precipitate formed at the interface of the added stock solutions that resolved during vortexing. After incubation for 20 h at room temperature, tubes were centrifuged at 11,000× *g* for 5 min at room temperature. The clear supernatant was removed by pipetting with a 1 mL pipet tip, and small amounts of the supernatant were removed by pipetting with a 0.1 mL pipet tip. The pellets were dried in air for several hours before being subjected to drying in a diaphragm vacuum (Welch, Dryfast Ultra, cat # 2032, Walktham, MA, USA) for 3 h. The pellet weight was determined by weighing the tubes with pellets on the fine balance and subtracting the empty tube weights. The data were processed and graphed in Microsoft Excel 16.101.

### 2.3. Measurements of pH

The reaction mixtures were generated at a 2 mL volume in 8.5 mL round-bottom tubes. This allowed the insertion of a regular glass electrode for pH determination. The pH meter was three-point calibrated (pH 4.0, 7.0, and 10.0) and specified for the pH range 0–14. To determine the amount of NaOH for neutralizing the mixtures of 100 mM of the phosphorus species and 200 mM MgCl_2_ or CaCl_2_, the same pH meter was used with a 50 mL buffer volume in a 100 mL glass beaker, and the pH was monitored during the addition of 1 M NaOH in small aliquots while stirring until neutrality was observed. The analogous volume of 1 M NaOH was added when preparing the 1 mL mixtures for the precipitation measurement. For precipitation experiments with the pH stabilized at pH 6.5, the mixtures received not only the NaOH solution but also a final concentration of 100 mM MOPS/NaOH pH 6.5, thereby first bringing the pH to 6.5 and then stabilizing it at pH 6.5. To estimate the three pK_A_ values of the acid corresponding to cTmp (Figure S1), 10 mmol of Na_3_cTmp was dissolved in 50 mL of water and 1 N HCl was added under stirring in aliquots from 0.1 mL to 1 mL while recording the pH after each addition. In addition, three mixtures with 100 mM Na_3_cTmp were generated with final concentrations of 50 mM, 150 mM, and 250 mM HCl, thereby bringing the fraction of cTmp to the first, second, and third half-protonation point. These pH values were determined in triplicate, and the averages were used as estimates. The values from the titration and the mixtures at half-titration points were within 0.2 pH units, and their average was used as an estimate given in Figure 1B.

## 3. Results

**Figure 2:**
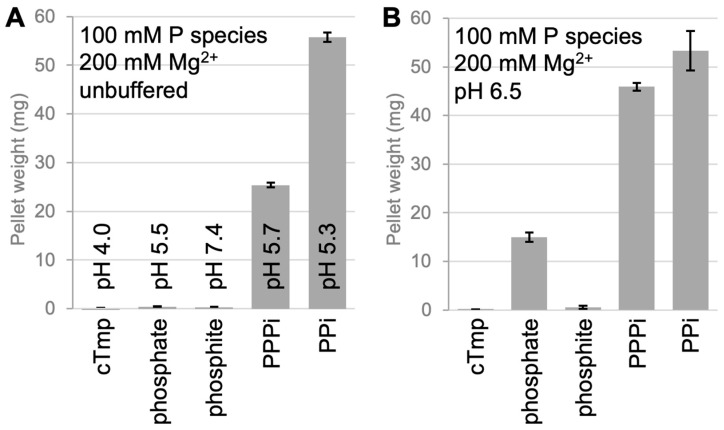
The precipitation of different phosphorus compounds at a concentration of 100 mM phosphorus and 200 mM Mg^2+^. Shown is the weight of precipitated material at a volume of 1 mL of the mixture after 20 h incubation, centrifugation, the removal of the supernatant, and the drying of the pellets. The name of the phosphorus compound is given for each species, with PPPi (linear tripolyphosphate) and PPi (pyrophosphate). (**A**) Precipitation after mixing the two components without the neutralization of buffering. (**B**) Precipitation after the components were mixed, neutralized with NaOH, and buffered at pH 6.5 with 100 mM MOPS/NaOH pH 6.5. Error bars are standard deviations of three experiments.

**Figure 3:**
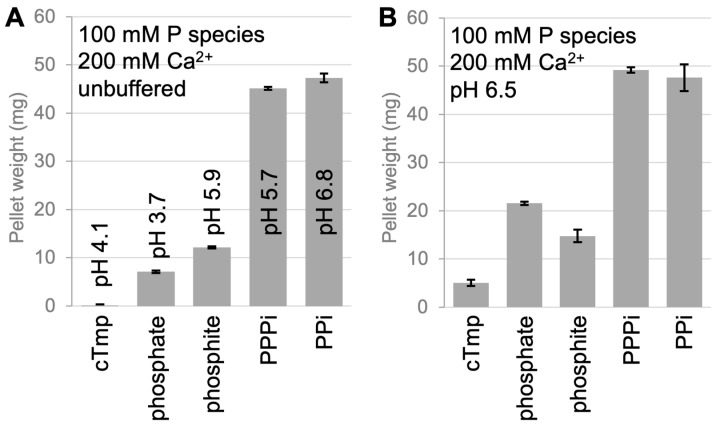
The precipitation of different phosphorus compounds at a concentration of 100 mM phosphorus and 200 mM Ca^2+^. Shown is the weight of precipitated material from a volume of 1 mL of the mixture after 20 h incubation, centrifugation, the removal of the supernatant, and the drying of the pellets. The name of the phosphorus compound is given for each species, with PPPi (linear triphosphate) and PPi (pyrophosphate). (**A**) Precipitation after mixing the two components without the neutralization of buffering. (**B**) Precipitation after the components were mixed, neutralized with NaOH, and buffered at pH 6.5 with 100 mM MOPS/NaOH pH 6.5. Error bars are standard deviations of three experiments.

To obtain semi-quantitative estimates of solubility and precipitation for different phosphorus compounds (Figure 1A,B) with prebiotically dominant divalent cations, we prepared the corresponding mixtures in a 1 mL volume, incubated for 20 h at room temperature, centrifuged for 5 min at 11,000× *g*, removed the supernatant, and dried and weighed the pellets (Figure 1C). These weights were plotted as a function of the concentration of the phosphorus species for the Mg^2+^ concentrations (Figure 2) and Ca^2+^ concentrations (Figure 3) from 10 mM to 1 M.

We first evaluated the solubility of condensed phosphates in the presence of Mg^2+^ because Mg^2+^ is required as a cofactor for many catalytic RNAs. Therefore, environments with Mg^2+^ and polyphosphates would fulfill two requirements for the rise of an RNA-based replicating system. No precipitation was observed with cTmp or phosphite (Figure 2), even with 200 mM cTmp or phosphite and 1 M Mg^2+^ (Figure S2). In contrast, linear tripolyphosphate (PPPi) and pyrophosphate (PPi) resulted in strong precipitation (Figure 2). The behavior of phosphate was pH0dependent: in an unbuffered reaction, where the chelation of Mg^2+^ by phosphate caused a drop in pH to pH 5.5, no precipitate was observed. In contrast, when the final reaction mixture was titrated to and stabilized at pH 6.5, phosphate led to significant precipitation with Mg^2+^. While local prebiotic environments may have had a pH of 5.5, the pH of most prebiotic environments was likely around 6.5 [34]. Therefore, the precipitation data shown in Figure 2B likely reflect the solubility of phosphorus species in most prebiotic environments.

The solubility of phosphorus compounds in the presence of Ca^2+^ is important for experimental models of prebiotic chemistry because the Ca^2+^ concentration was likely the dominant divalent cation in Earth’s prebiotic oceans, around 200 mM [34]. In our experiments, the solubility of condensed phosphates with 200 mM Ca^2+^ led to similar but more pronounced results than Mg^2+^. In unbuffered solution with pH values dropping due to chelation (Figure 3A), no precipitation was observed with cTmp, little precipitation with phosphate and phosphite, and strong precipitation with linear tripolyphosphate and pyrophosphate. When the pH was corrected to and stabilized at pH 6.5 (Figure 3B), small amounts of precipitates were found for cTmp, medium precipitation resulted from phosphite and phosphate, and strong precipitation was observed for linear tripolyphosphate and pyrophosphate. The precipitation of phosphite with Ca^2+^ is visible in calcium phosphite deposits in 3.5-billion-year-old marine sediments, which suggested that phosphite was available on early Earth in areas of the late Hadean ocean [36].

**Figure 4:**
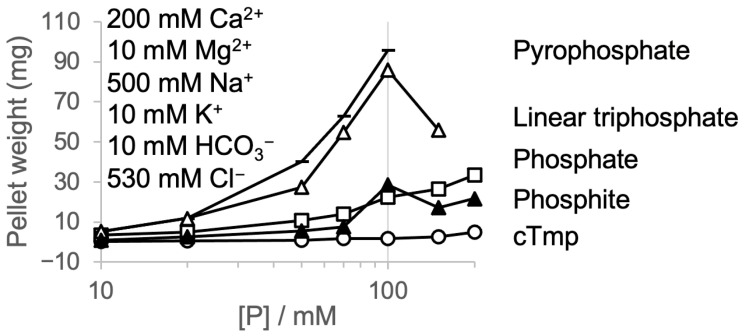
The precipitation of different phosphorus species in a mixture modeling prebiotic ocean water. The weight of the pellets is plotted as a function of the phosphorus species’ concentration. The concentrations of the ions in the mixture are given in the insert. The data series are labeled on the right, with pyrophosphate (the horizontal line), linear triphosphate (the empty triangle), phosphate (the empty square), phosphite (the filled triangle), and cyclic trimetaphosphate (the circle). The solutions were unbuffered, making these series comparable to the results shown in Figure S3.

To model the environment of prebiotic seawater, we used the values obtained for Earth’s oceans of four billion years ago [34] and approximated them with 200 mM Ca^2+^, 10 mM Mg^2+^, 500 mM Na^+^, 10 mM K^+^, 10 mM HCO^3−^ and 530 mM Cl^−^. The incubation of this mixture with the different phosphorus species at increasing concentrations (Figure 4) resulted in precipitation similar to the precipitation behavior with Ca^2+^ (Figure S3), which is unsurprising given that Ca^2+^ is the dominant divalent cation in the ‘prebiotic ocean’ mixture. Again, cyclic trimetaphosphate was the most soluble phosphorus compound. Together, these results illustrate the extraordinary solubility of cyclic trimetaphosphate under conditions relevant for early life scenarios.

## 4. Discussion

This study evaluated the solubility of several phosphorus compounds with relevance to origins-of-life scenarios in the presence of Mg^2+^ and Ca^2+^ divalent cations. The results showed that cyclic trimetaphosphate (cTmp) remained soluble over the complete scale of tested Mg^2+^ concentrations up to 1 M and showed little precipitation with 200 mM Ca^2+^ and at model conditions for the prebiotic ocean with 200 mM Ca^2+^ and 10 mM Mg^2+^. In contrast, pyrophosphate and linear tripolyphosphate showed strong precipitation, even below 100 mM Mg^2+^ and Ca^2+^ concentrations. The solubility of orthophosphate depended on the pH, with lower solubility than cTmp and phosphite under prebiotically relevant conditions.

Why are cTmp complexes with divalent cations so soluble and the complexes of other condensed polyphosphates are not? At any prebiotically relevant pH, cTmp carries three negatively charged oxygens because the three corresponding pK_A_ values are below pH 2 (Figure 1A and Figure S1). Two of these oxygen anions can tightly coordinate one divalent cation [37]. The dissociation constants of the Ca^2+^/cTmp^3−^ complex is in the range of 0.3 mM in a very dilute solution [29] and weakens with increasing monovalent ion concentrations to a K_D_ of about 5 mM at 50 mM Na^+^ and 15 mM at 200 mM Na^+^ [37]. Only the 1:1 complex appears to form at millimolar concentrations [37], which means that the complex carries one negative charge under dilute conditions, which helps solubilize the complex in water. A second binding event that occurs to form the charged [Ca_2_cTmp]^+^ complex is negligible when the components are at a 10 mM concentration (Davies & Monk (1949) https://doi.org/10.1039/JR9490000413 [29], Kura et al. (1974) https://doi.org/10.1016/0022-1902(74)80631-7 [37]). We did not find studies on a second divalent metal binding event at high divalent cation concentrations, except a possible second binding event when free Mg^2+^ is around 100 mM in excess of cTmp (Moretti & Muller (2014) https://doi.org/10.1093/nar/gkt1405 [20]). The resulting complex [Mg_2_/cTmp]^+^ would have a single positive charge, which would help keep the complex soluble. This scenario is complicated in a mixed Mg^2+^/Ca^2+^ solution by the observation that Mg^2+^ has dramatic effects on the kinetics of calcium phosphate formation (Boskey & Posner 1974, https://doi.org/10.1016/0025-5408(74)90169-X [38]). A detailed experimental analysis of the complexes present at high divalent metal ion concentrations is still outstanding.

In contrast, pyrophosphate carries one hydroxyl group with a pK_A_ around 7 at each terminal phosphate (Figure 1), which forms with Mg^2+^ the very stable Mg_2_P_2_O_7_ [39,40]. Similarly, linear tripolyphosphate (and all linear polyphosphates in general) also have two terminal hydroxyls with a pK_A_ around 7, and the strong electrostatic crosslinking with divalent cations can mediate the precipitation of polyphosphate chains [41]. While longer linear and cyclic condensed phosphates than those studied here have been isolated and studied (for an early overview, see [27]), their yield in prebiotically plausible syntheses is much lower, especially since long polyphosphates tend to form cyclic trimetaphosphate and other short phosphate species in contact with water [33]. Therefore, this study’s focus on orthophosphate, pyrophosphate, linear tripolyphosphate, cyclic trimetaphosphate, and phosphite covers the dominant species in a likely prebiotic scenario.

Together, these results suggest that in a prebiotically plausible scenario where condensed phosphates are formed, the presence of Mg^2+^ and Ca^2+^ ions in concentrations similar to the prebiotic ocean would precipitate most phosphorus species while leaving a large fraction of cTmp and phosphite in the supernatant.

What is the benefit of being soluble? For lab experiments, one benefit of soluble substrates is an easier set up and analysis of a model system in vitro [20,21,30,31,32,42,43]. Nevertheless, it is possible that the earliest self-replicating and evolving molecular systems did not function in free solution, in lipid vesicle compartments [11] or in coacervate compartments [44] but on the interfaces of minerals such as on iron sulfide [45], in rock pores with heat gradients [12] or in sediments partially composed of ‘insoluble’ material. However, an advantage of fully dissolved metabolites is that their reaction rates are orders of magnitude faster than solid–solid reactions [46]. Therefore, the good solubility of cTmp in the presence of divalent cations is an important factor that makes cTmp an excellent candidate for a primordial metabolite. In addition, this study also confirmed the relatively high solubility of phosphite in solutions rich in divalent metals (Figure 2, Figure 3 and Figure 4). This is important for possible prebiotic reaction pathways generating cTmp from phosphite in the presence of pyrophosphate and mild oxidation sources [17]. Given that the precipitation of phosphite is more pronounced with Ca^2+^ than Mg^2+^ (compare Figure 2 and Figure 3), it may be useful to model prebiotic reactions in the presence of calcium.

Cyclic trimetaphosphate has several additional advantages regarding its possible involvement as an energy source for early life forms. In addition to being the best polyphosphorylation reagent of all polyphosphates [27] and reacting with nucleoside 5′-hydroxyl groups to generate NTPs with and without catalysts [20,21,30,31,32], cTmp is a promising energy source for RNA-based life forms because the availability of cTmp at low concentration could have allowed chemical pathways to begin operating at slow rates, and the large rate increases upon catalysis place a large evolutionary advantage on the emergence of ribozymes to catalyze this reaction. The ability of ribozymes to utilize ribozyme-generated NTPs [21] could have formed the core of a small nucleotide-based metabolism that could have supported early RNA-dominated life forms

## Data Availability

The original contributions presented in this study are included in the article/Supplementary Material. Further inquiries can be directed to the corresponding author.

## Supplementary Materials

The following supporting information can be downloaded at https://www.mdpi.com/article/10.3390/life16010184/s1, Figure S1: Estimation of the pK_A_ values of cTmp by titration with 1N HCl; Figure S2: Graphs of phosphate species incubated with Mg^2+^; Figure S3: Graphs of phosphate species incubated with Ca^2+^; Figure S4: Tube images of phosphate species and Mg^2+^; Figure S5: Tube images of phosphate species and Ca^2+^; Figure S6: Tube images of phosphate species under seawater model conditions.
