# Supplementary material for: The Exceptional Solubility of Cyclic Trimetaphosphate in the Presence of Mg2+ and Ca2+
Source: Life (Basel). 2026 Jan 22;16(1):184. doi: 10.3390/life16010184 (PMC12842746; doi:10.3390/life16010184)
Supplement: Supplementary file 1 [file life-16-00184-s001.zip › life-3936093-supplementary.pdf]

Supplementary Figures

The Exceptional Solubility of cyclic Trimetaphosphate in the Presence of Mg^{2+} and Ca^{2+}

Megan G. Bachant and Ulrich F. Müller

Content **page**

Figure S1: Estimation of the pK_A values of cTmp by titration with 1N HCl 2

Figure S2: Graphs of Phosphate species and Mg^{2+} 3

Figure S3: Graphs of Phosphate species and Ca^{2+} 4

Figure S4: Tube images of phosphate species and Mg^{2+} 5

Figure S5: Tube images of phosphate species and Ca^{2+} 12

Figure S6: Tube images of phosphate species under seawater model conditions (100 mM phosphate species and prebiotic seawater solution using 200 mM Ca^{2+}, 10 mM Mg^{2+}, 10 mM sodium bicarbonate, 100 mM Na^{+},10 mM K^{+}) 19

Figure S1

A

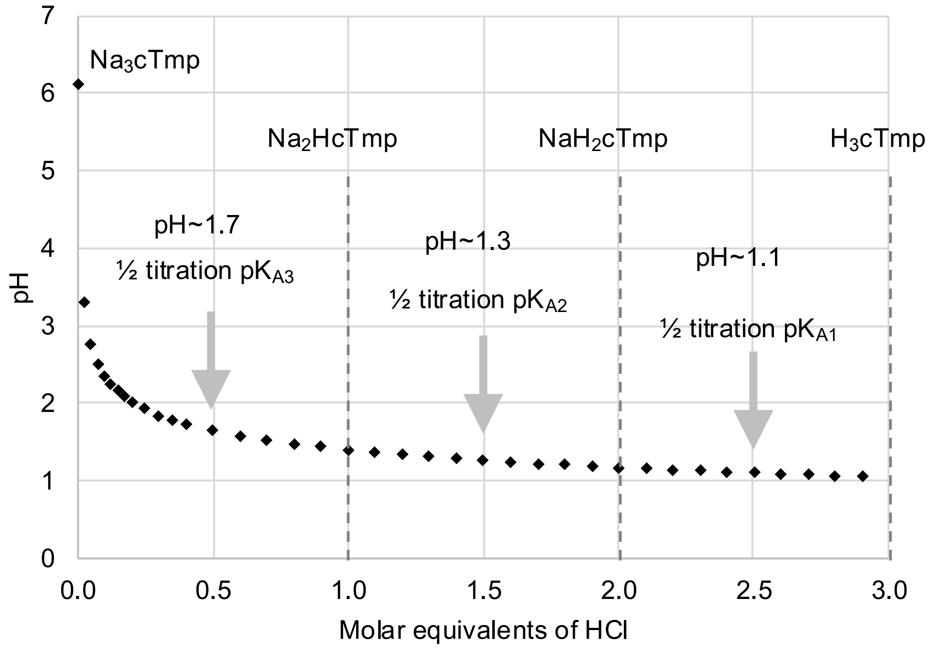

B

$[Na_3cTmp]$	[HCl]	pK_A	pH
100 mM	50 mM	pK_{A3}	1.88 ± 0.02
100 mM	150 mM	pK_{A2}	1.43 ± 0.01
100 mM	250 mM	pK_{A1}	1.24 ± 0.04

Figure S1. Estimation of the pK_As for trimetaphosphopric acid. **(A)** Titration of 100 mL 100mM Na_3cTmp with 1N HCl. The pH values are plotted as function of the molar equivalent of HCl added. As estimates of the pK_A values, the pH values at the half titration points for each of the three protonation steps are shown in blue. **(B)** pH measurement at fixed volumes and concentrations of 100 mM Na_3cTmp with 0.5 molar equivalent (50mM), 1.5 molar equivalents (150 mM), and 2.5 molar equivalents (250mM) of HCl. Given errors are standard deviations from three technical replicates. All measurements were done using a glass electrode with 3-point calibration and specification between pH 0 – 14.

Figure S2

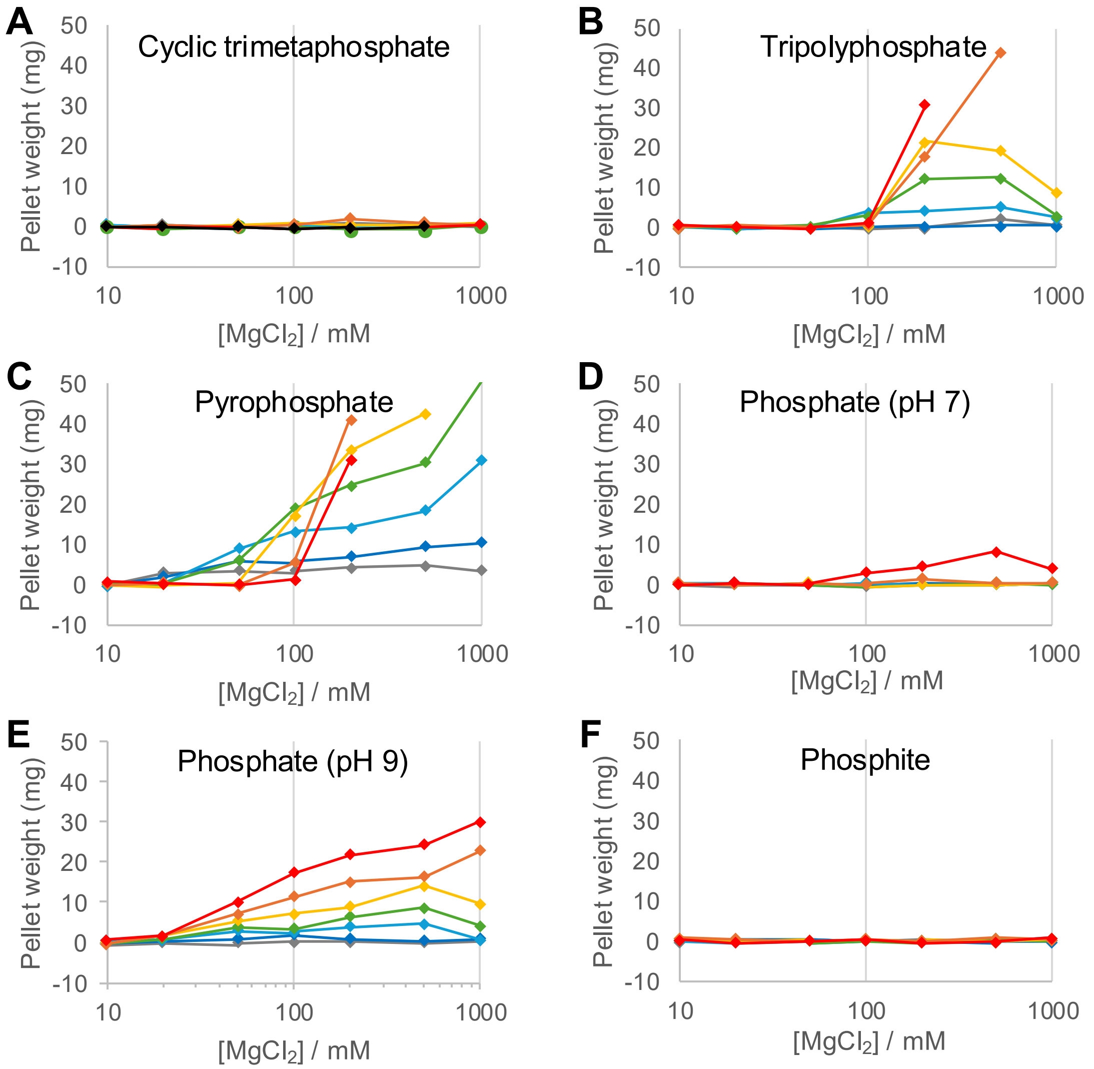

Figure S2: Precipitation of different phosphorus compounds at increasing concentration of Mg^{2+}. The weight of dried pellets is plotted as a function of Mg^{2+} concentration. The colors of the data reflect the concentration of the phosphorus compounds, with black (500 mM, only for cTmp), red (200 mM), orange (150 mM), yellow (100 mM), green (70 mM), light blue (50mM), dark blue (20 mM) and gray (10mM). Several data series are truncated at high concentration due to the limited solubility of the phosphorus compound in water. The name of the phosphorus compound is given for each species, with **(A)** cTmp, **(B)** linear tripolyphosphate, **(C)** pyrophosphate, **(D)** orthophosphate at pH 7 (50% dihydrogen phosphate and 50% hydrogenphosphate), **(E)** orthophosphate at pH 9 (10% dihydrogen phosphate and 90% hydrogenphosphate), and **(F)** phosphite / H-phosphonate.

Figure S3

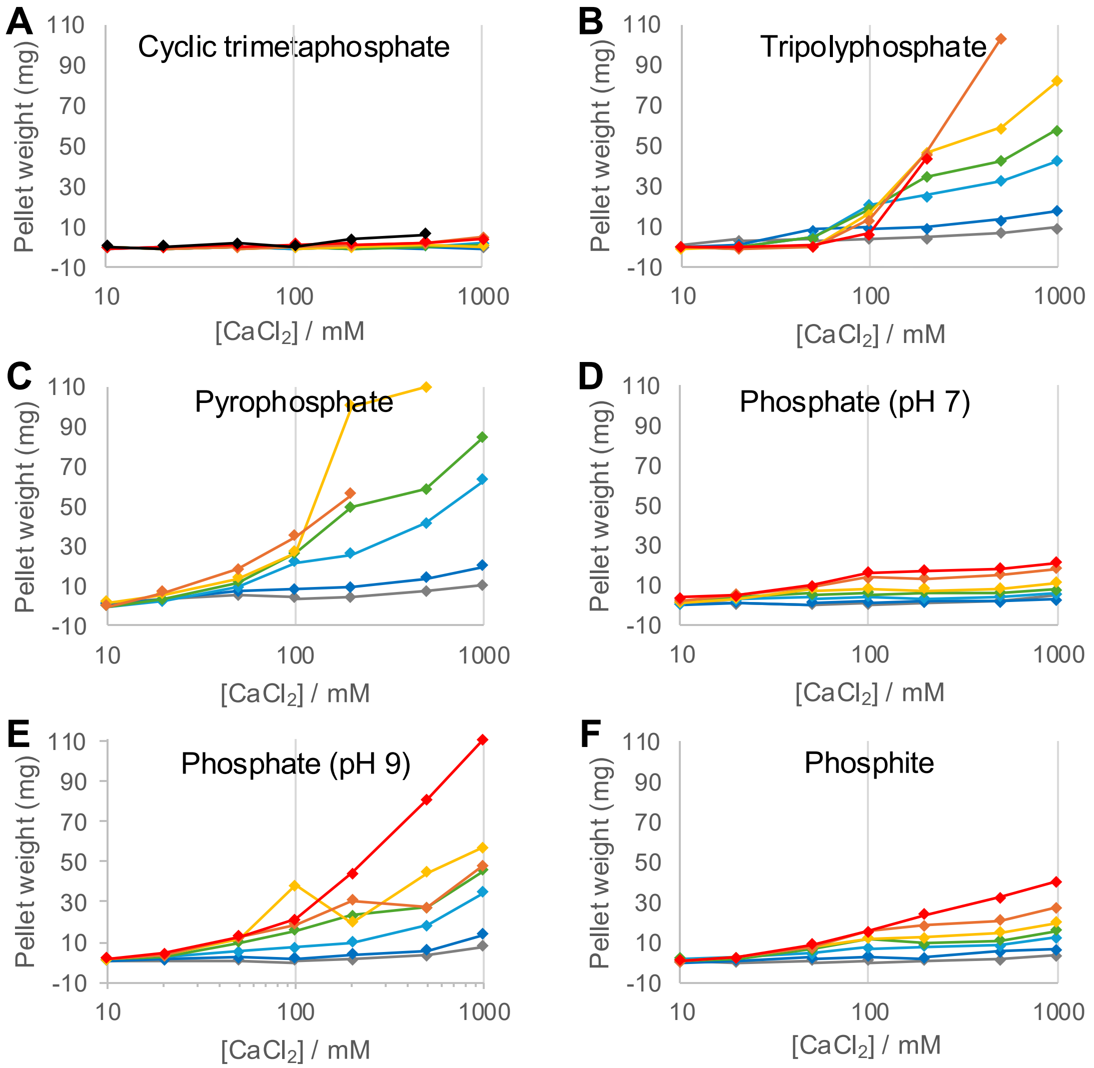

Figure S3: Precipitation of different phosphorus compounds at increasing concentration of Ca^{2+}. The weight of dried pellets is plotted as a function of Ca^{2+} concentration. The colors of the data reflect the concentration of the phosphorus compounds, with black (500 mM, only for cTmp), red (200 mM), orange (150 mM), yellow (100 mM), green (70 mM), light blue (50mM), dark blue (20 mM) and gray (10mM). Several data series are truncated at high concentration due to the limited solubility of the phosphorus compound in water. The name of the phosphorus compound is given for each species, with **(A)** cTmp, **(B)** linear triphosphate, **(C)** pyrophosphate, **(D)** orthophosphate (50% dihydrogen phosphate and 50% hydrogenphosphate), **(E)** orthophosphate (10% dihydrogen phosphate and 90% hydrogenphosphate), and **(F)** phosphite / H-phosphonate. The y-axis maximum was set to a higher value than the plots with Mg^{2+} because the larger pellets had a higher overall mass, consistent with the higher atomic weight of Ca^{2+} (40 g/mol) compared toMg^{2+} (23 g/mol)

Figure S4

A.

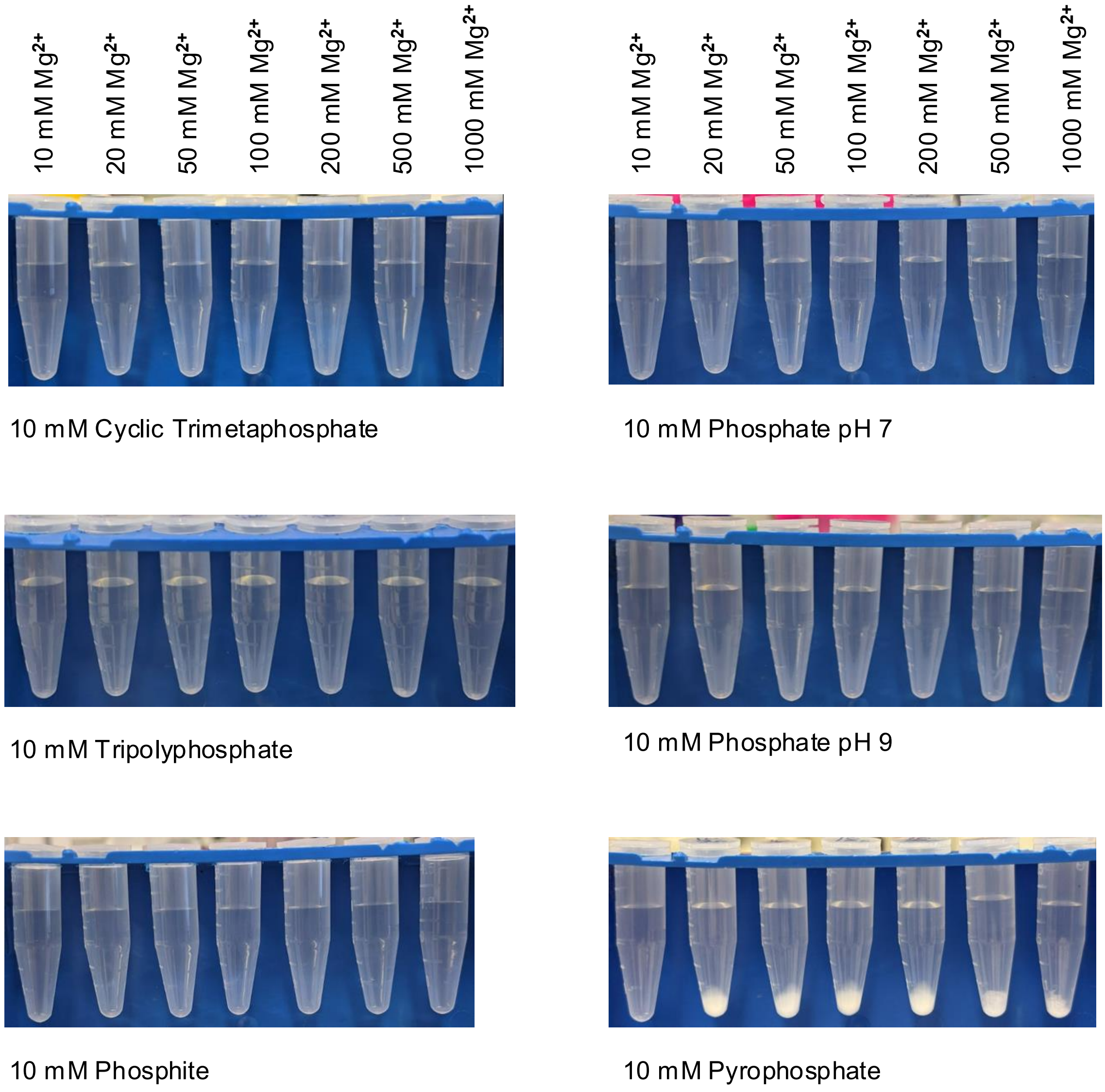

(A.) 1 mL total reaction volumes containing 10 mM of each phosphate species and varied Mg^{2+} concentrations, incubated overnight and centrifuged at 11000 g for 5 minutes.

Figure S4

B.

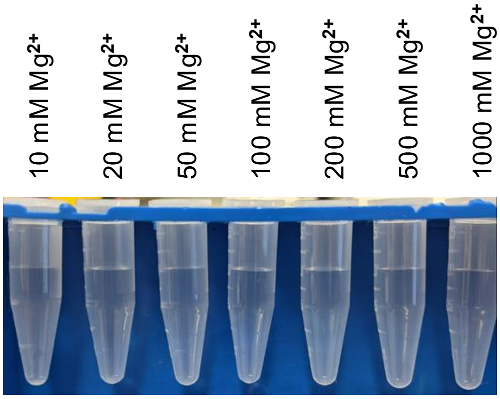

20 mM Cyclic Trimetphosphate

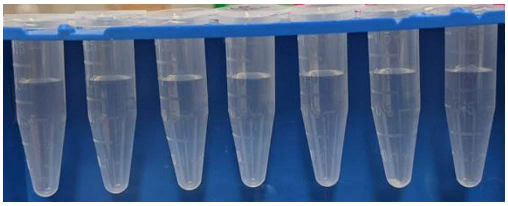

20 mM Tripolyphosphate

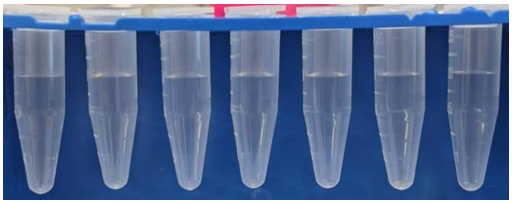

20 mM Phosphite

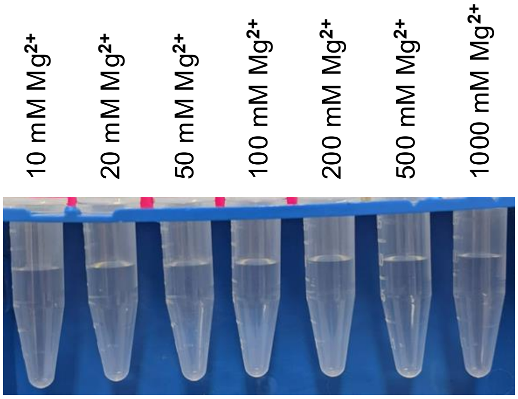

20 mM Phosphate pH 7

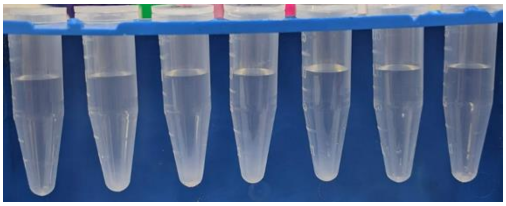

20 mM Phosphate pH 9

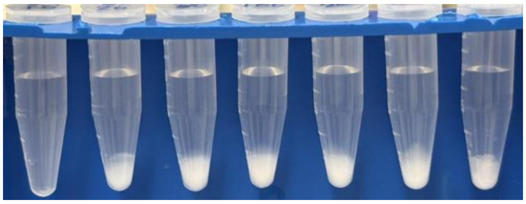

20 mM Pyrophosphate

(B.) 1 mL total reaction volumes containing 20 mM of each phosphate species and varied Mg^{2+} concentrations, incubated overnight and centrifuged at 11000 g for 5 minutes.

Figure S4

C.

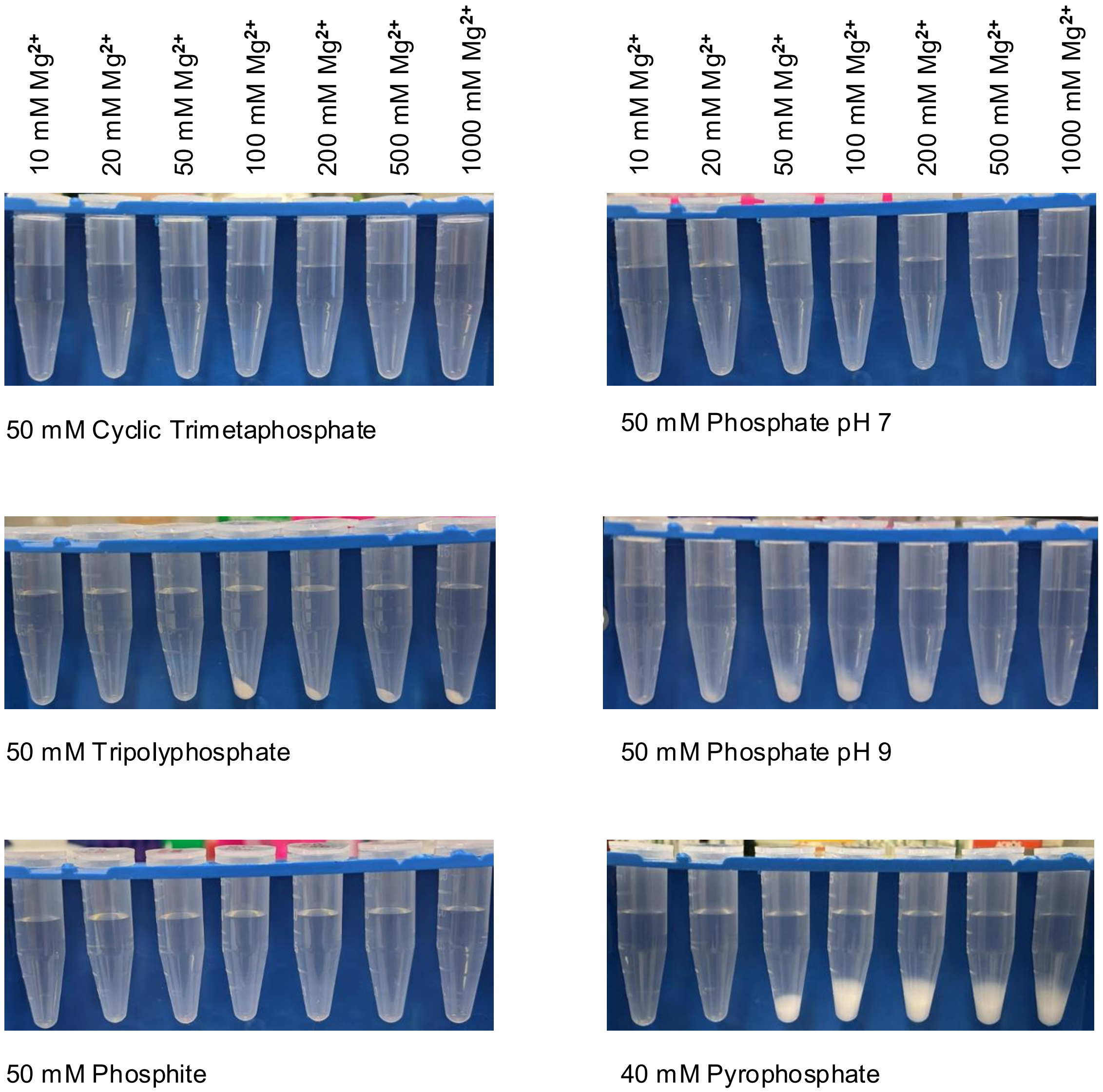

(C.) 1 mL total reaction volumes containing 50 mM, 40 mM in the case of pyrophosphate, of each phosphate species and varied Mg^{2+} concentrations, incubated overnight and centrifuged at 11000 g for 5 minutes.

Figure S4

D.

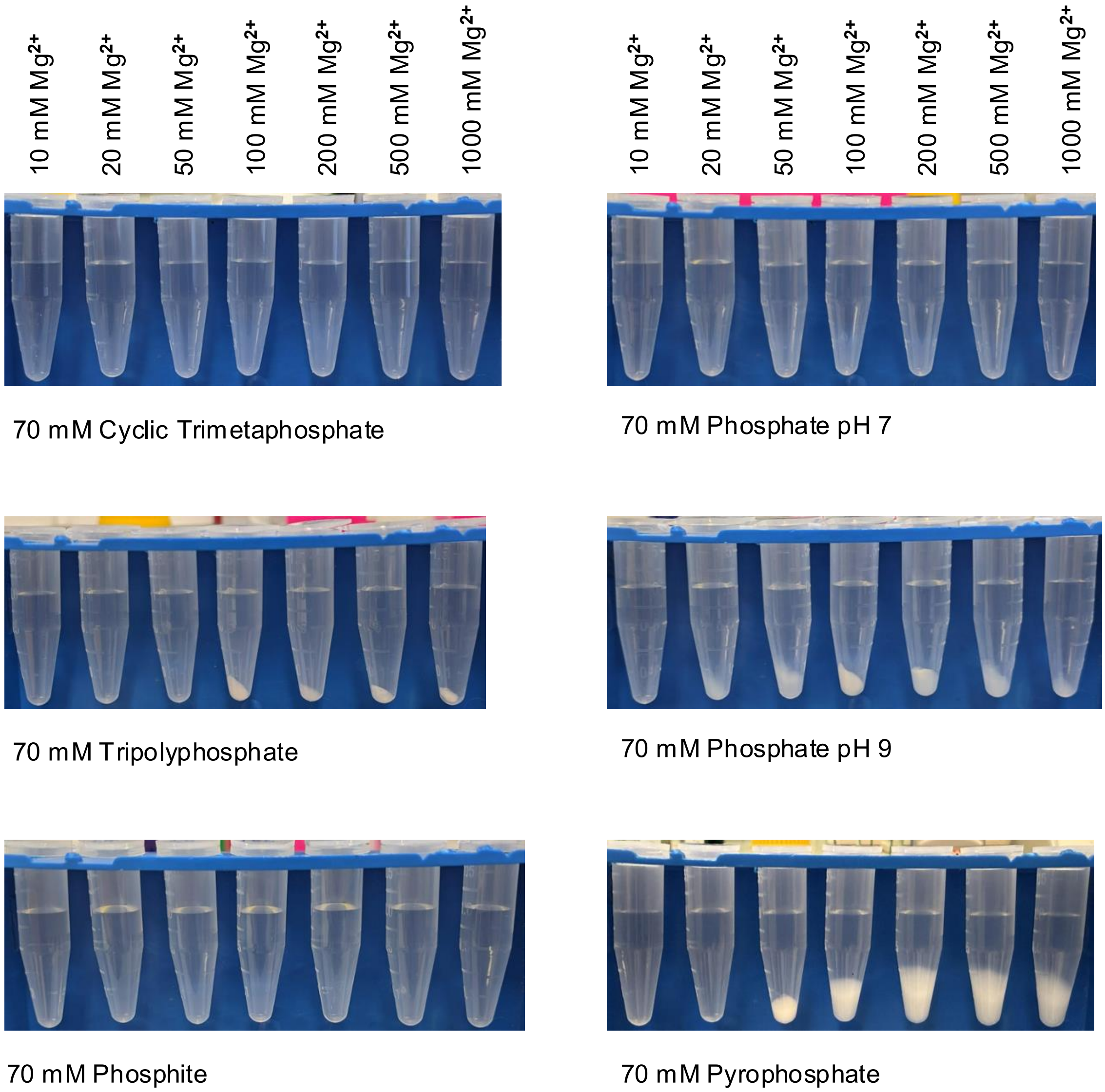

(D.) 1 mL total reaction volumes containing 70 mM of each phosphate species and varied Mg^{2+} concentrations, incubated overnight and centrifuged at 11000 g for 5 minutes.

Figure S4

E.

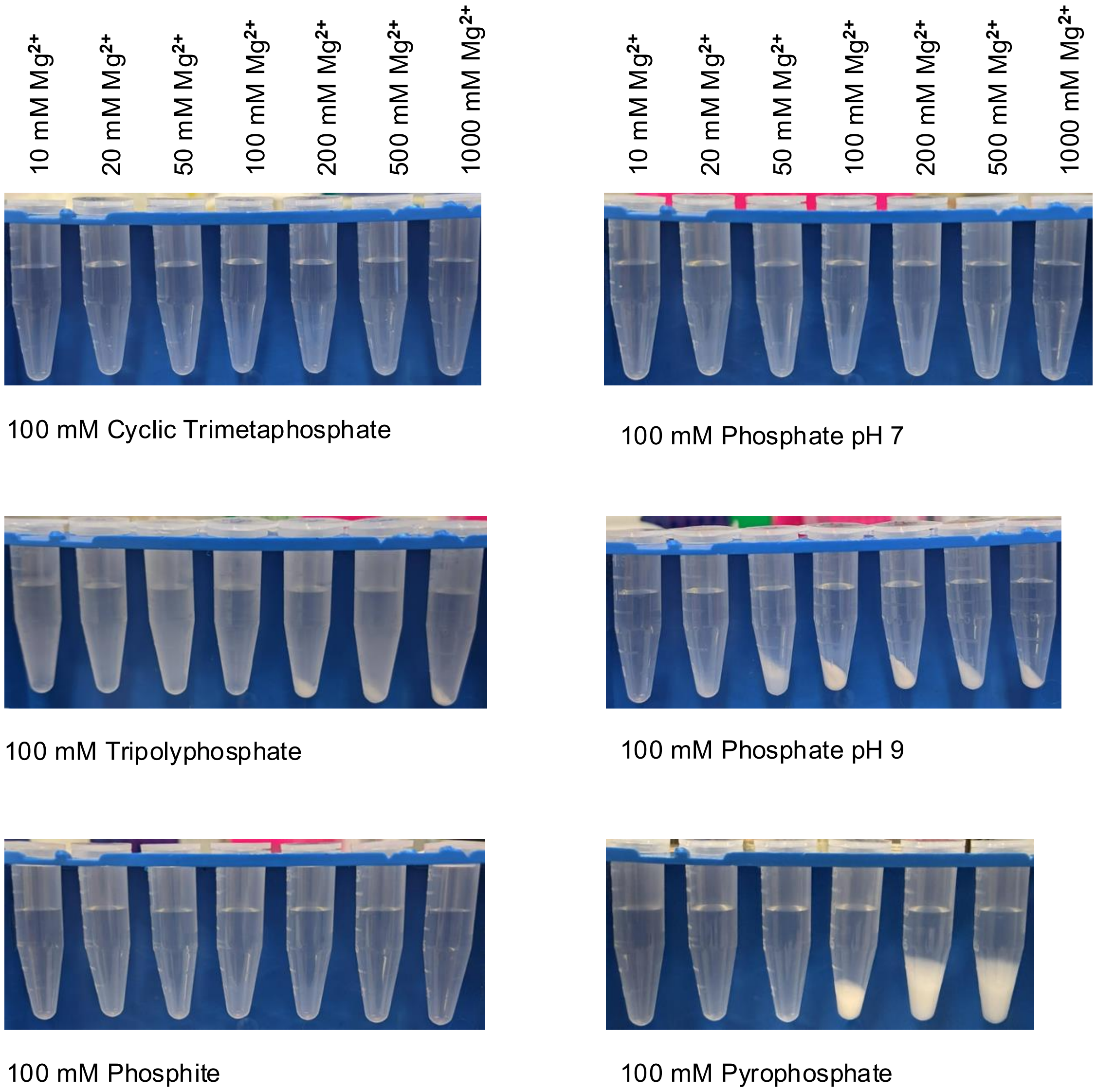

(E.) 1 mL total reaction volumes containing 100 mM of each phosphate species and varied Mg^{2+} concentrations, incubated overnight and centrifuged at 11000 g for 5 minutes. Due to solubility and volume constraints 1000 mM Mg^{2+} was not included in the case of pyrophosphate.

Figure S4

F.

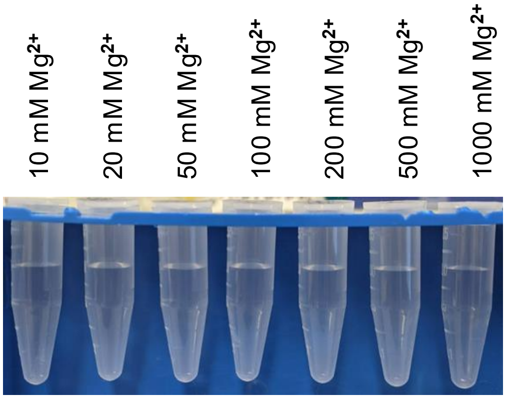

150 mM Cyclic Trimetaphosphate

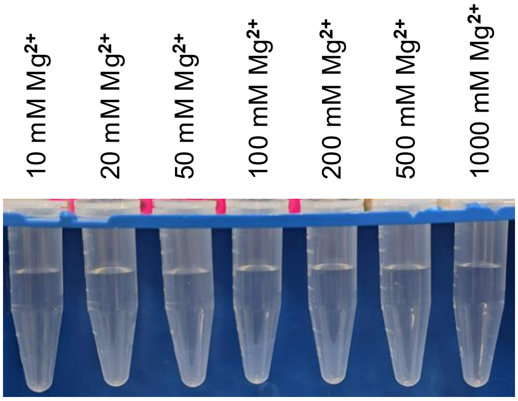

150 mM Phosphate pH 7

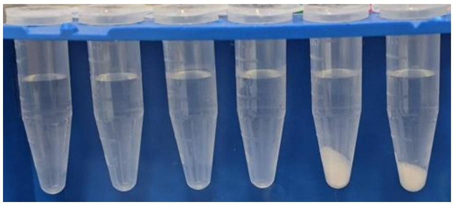

150 mM Tripolyphosphate

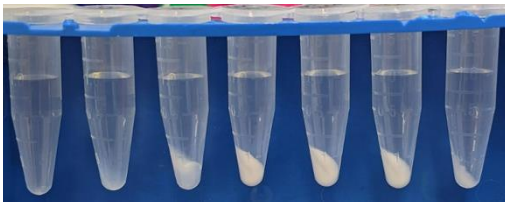

150 mM Phosphate pH 9

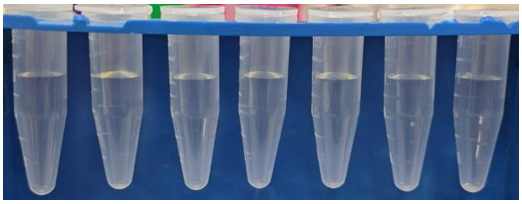

150 mM Phosphite

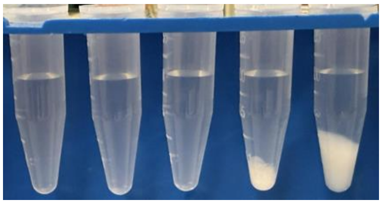

150 mM Pyrophosphate

(F.) 1 mL total reaction volumes containing 150 mM of each phosphate species and varied Mg^{2+} concentrations, incubated overnight and centrifuged at 11000 g for 5 minutes. Due to solubility and volume constraints 1000 mM Mg^{2+} was not included in the case of tripolyphosphate, and 500 and 1000 mM Mg^{2+} were not included in the case of pyrophosphate.

Figure S4

G.

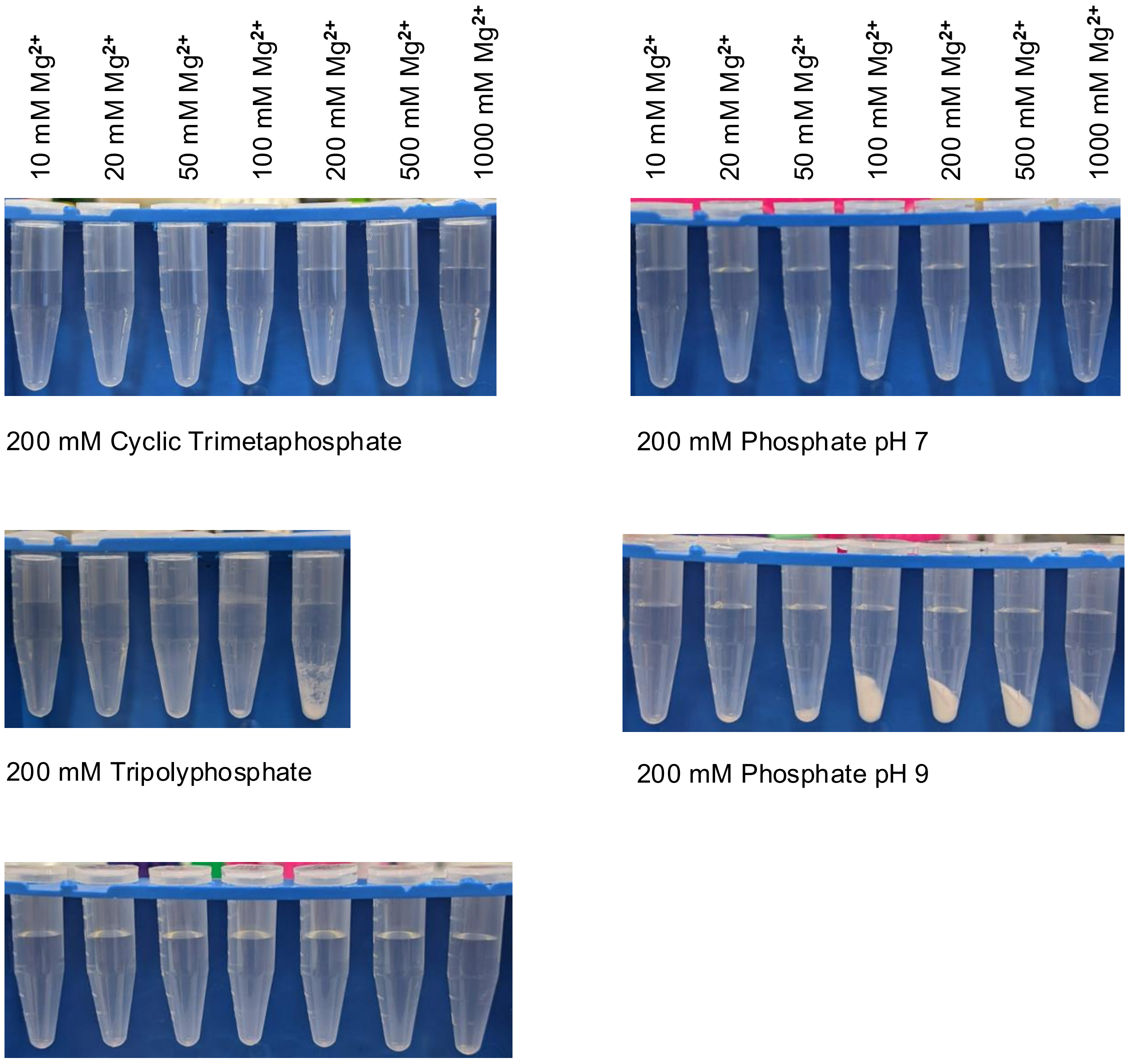

(G.) 1 mL total reaction volumes containing 200 mM of each phosphate species and varied Mg^{2+} concentrations, incubated overnight and centrifuged at 11000 g for 5 minutes. Due to solubility and volume constraints 500 and 1000 mM Mg^{2+} were not included in the case of tripolyphosphate. Pyrophosphate is not soluble in water at 200 mM and was not included.

Figure S5

A.

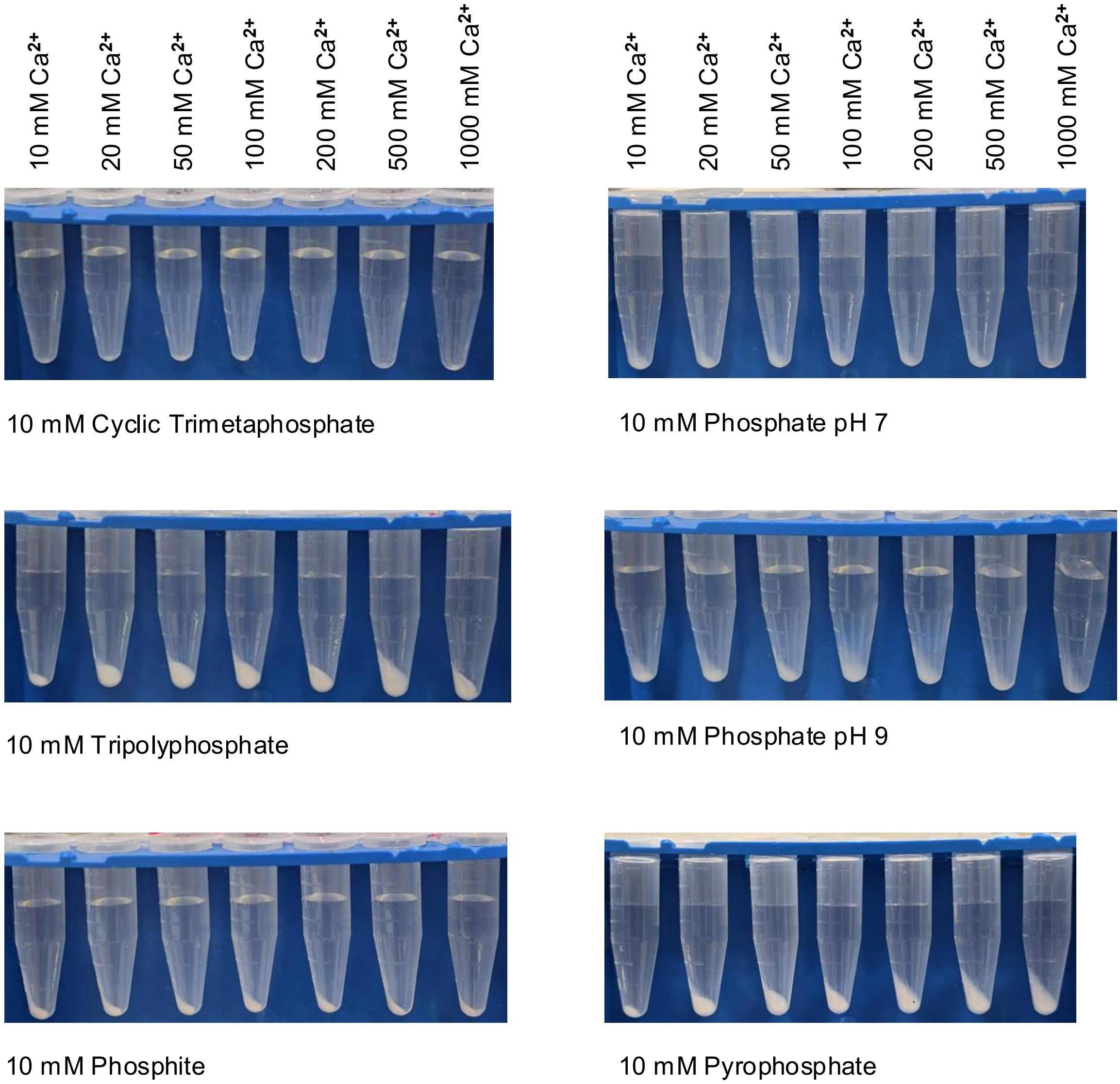

(A.) 1 mL total reaction volumes containing 10 mM of each phosphate species and varied Ca^{2+} concentrations, incubated overnight and centrifuged at 11000 g for 5 minutes.

Figure S5

B.

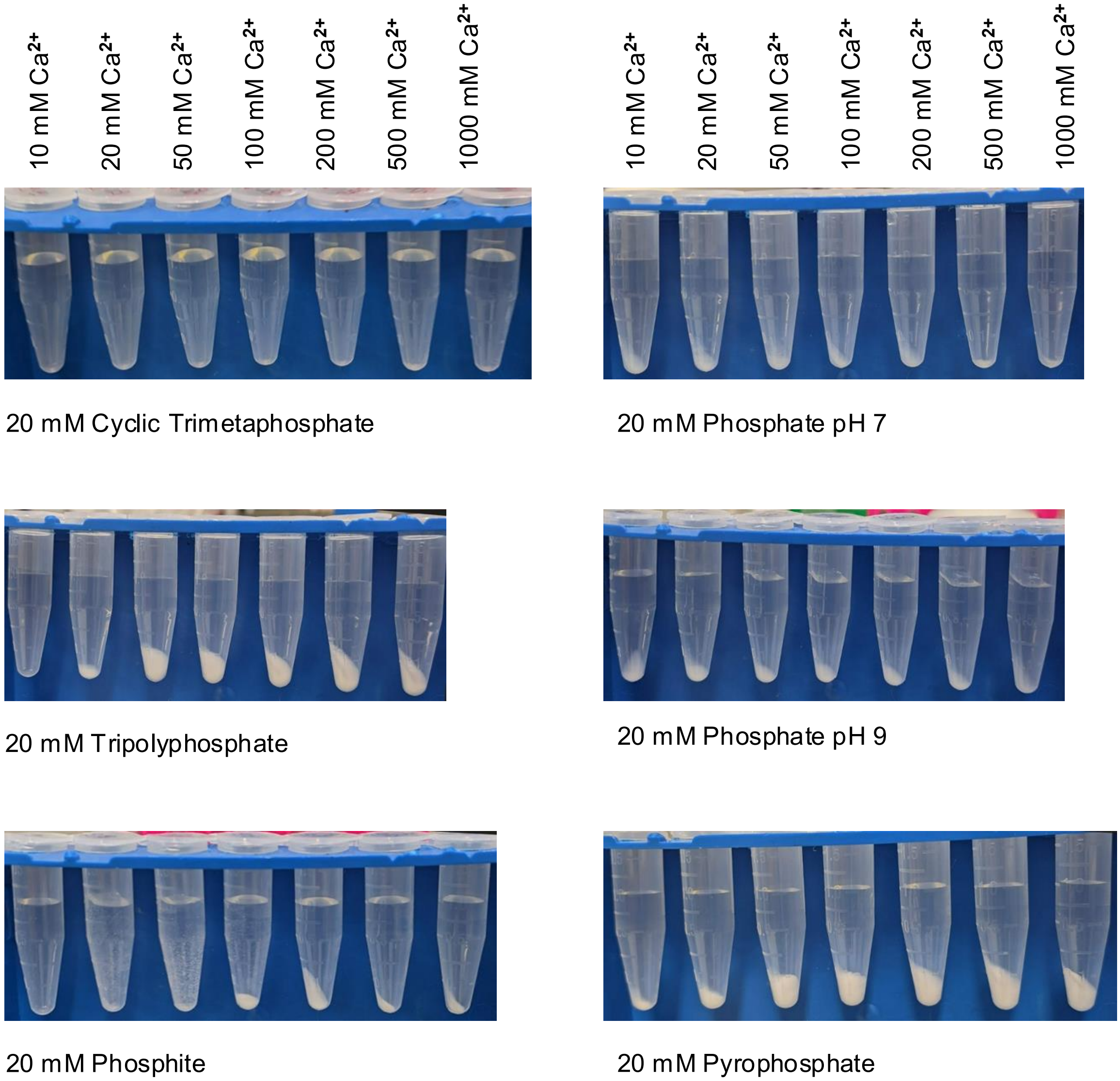

(B.) 1 mL total reaction volumes containing 20 mM of each phosphate species and varied Ca^{2+} concentrations, incubated overnight and centrifuged at 11000 g for 5 minutes.

Figure S5

C.

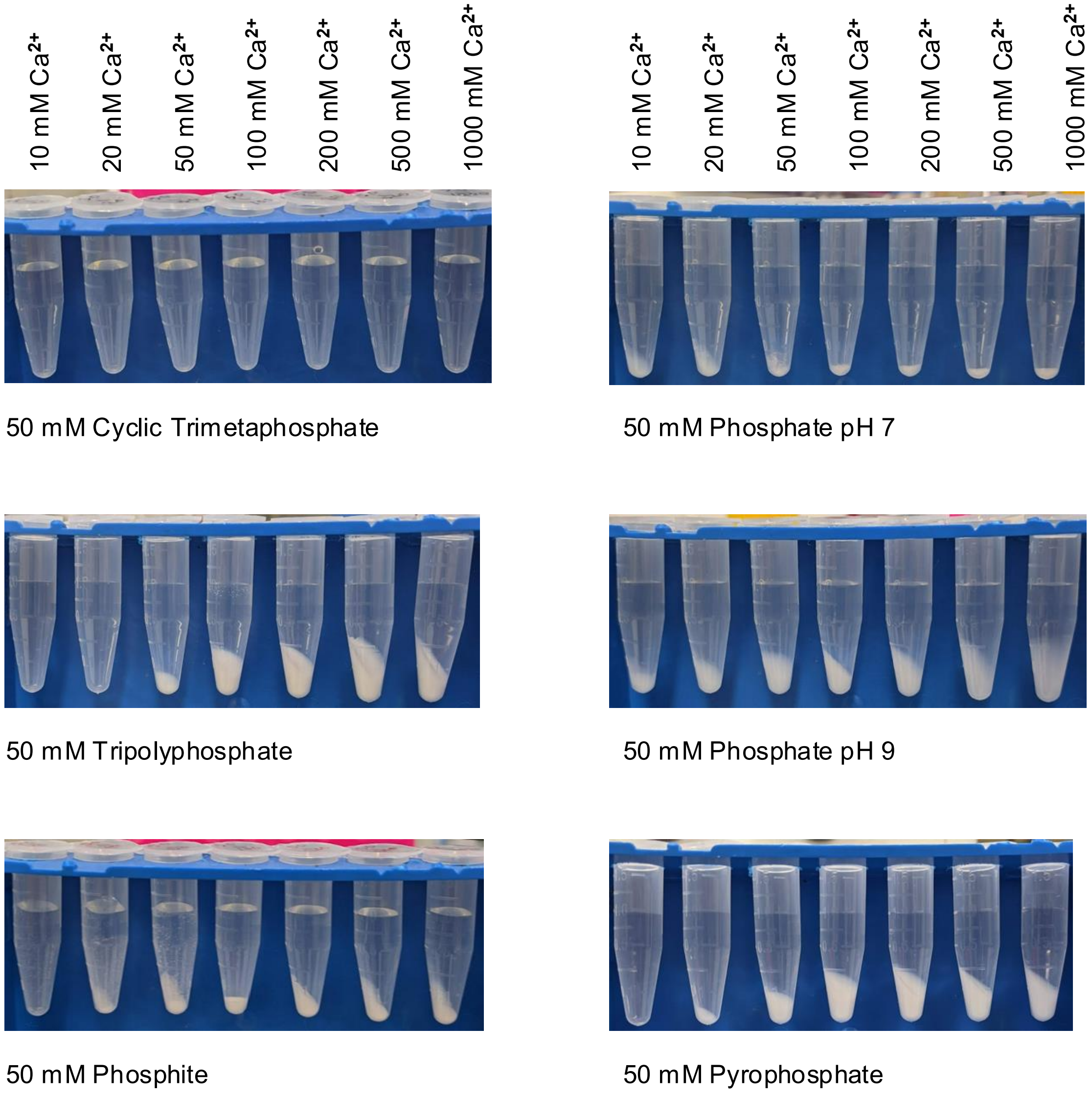

(C.) 1 mL total reaction volumes containing 50 mM of each phosphate species and varied Ca^{2+} concentrations, incubated overnight and centrifuged at 11000 g for 5 minutes.

Figure S5

D.

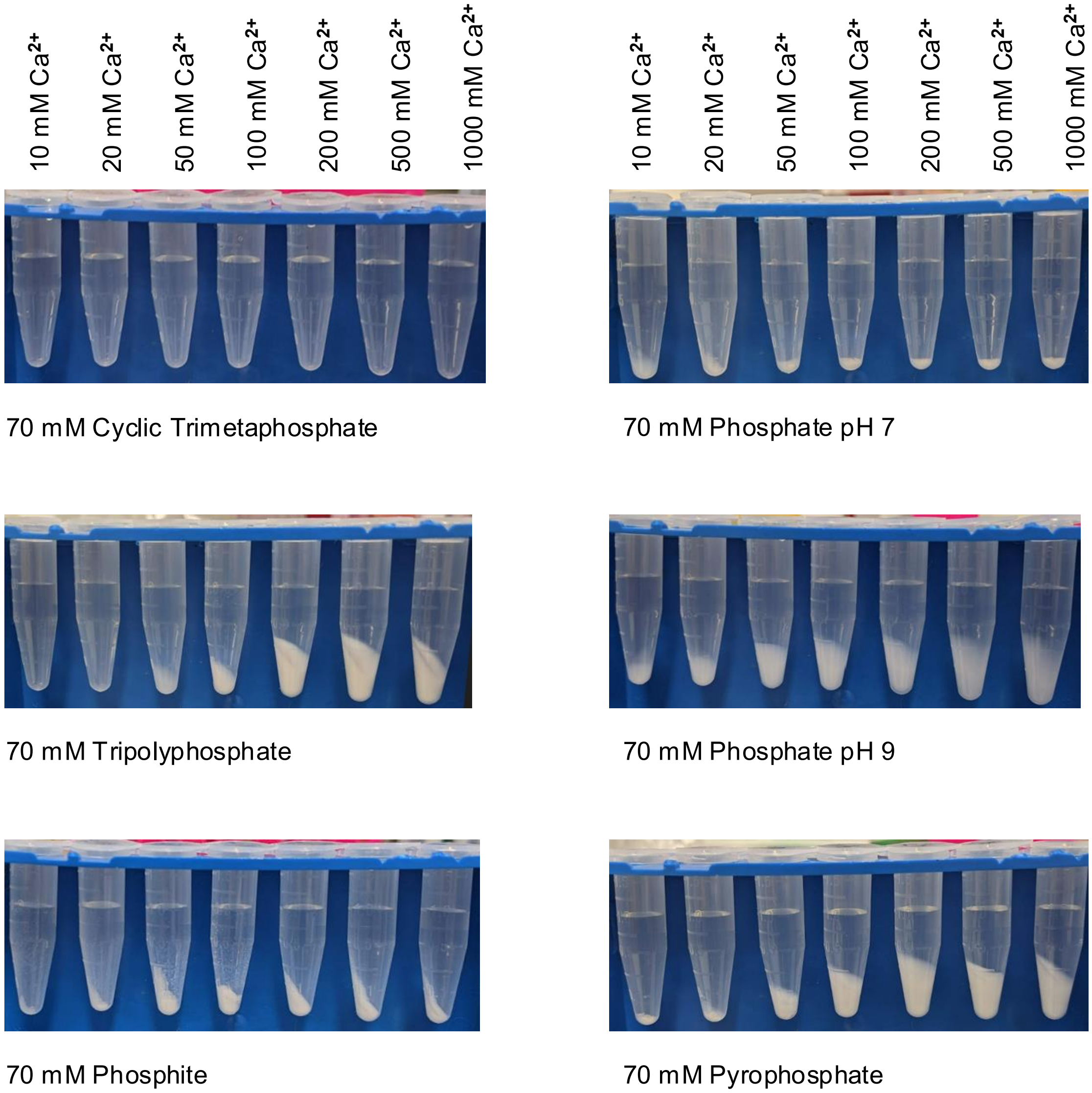

(D.) 1 mL total reaction volumes containing 70 mM of each phosphate species and varied Ca^{2+} concentrations, incubated overnight and centrifuged at 11000 g for 5 minutes.

Figure S5

E.

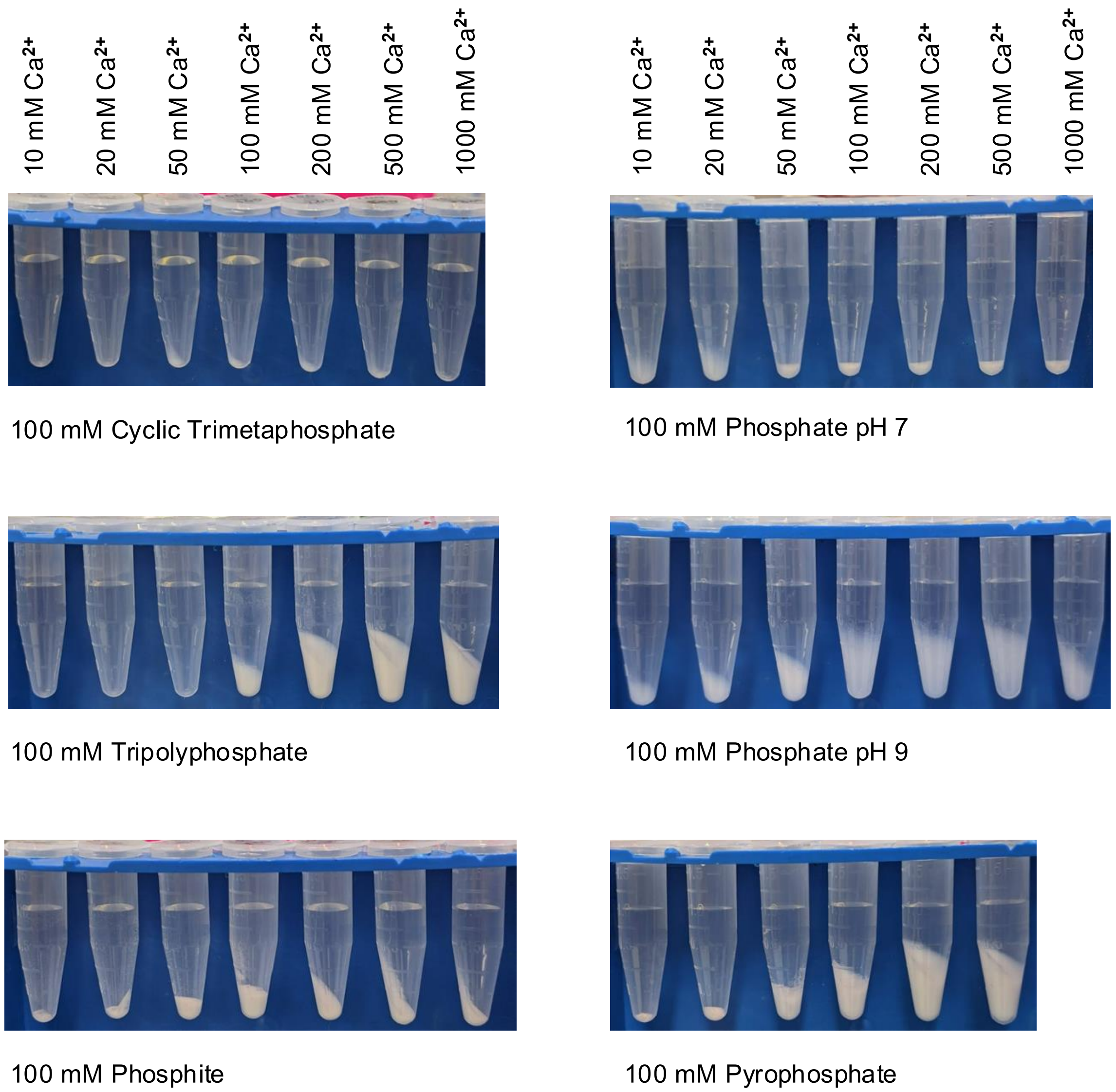

(E.) 1 mL total reaction volumes containing 100 mM of each phosphate species and varied Ca^{2+} concentrations, incubated overnight and centrifuged at 11000 g for 5 minutes. Due to solubility and volume constraints 1000 mM Ca^{2+} was not included in the case of pyrophosphate.

Figure S5

F.

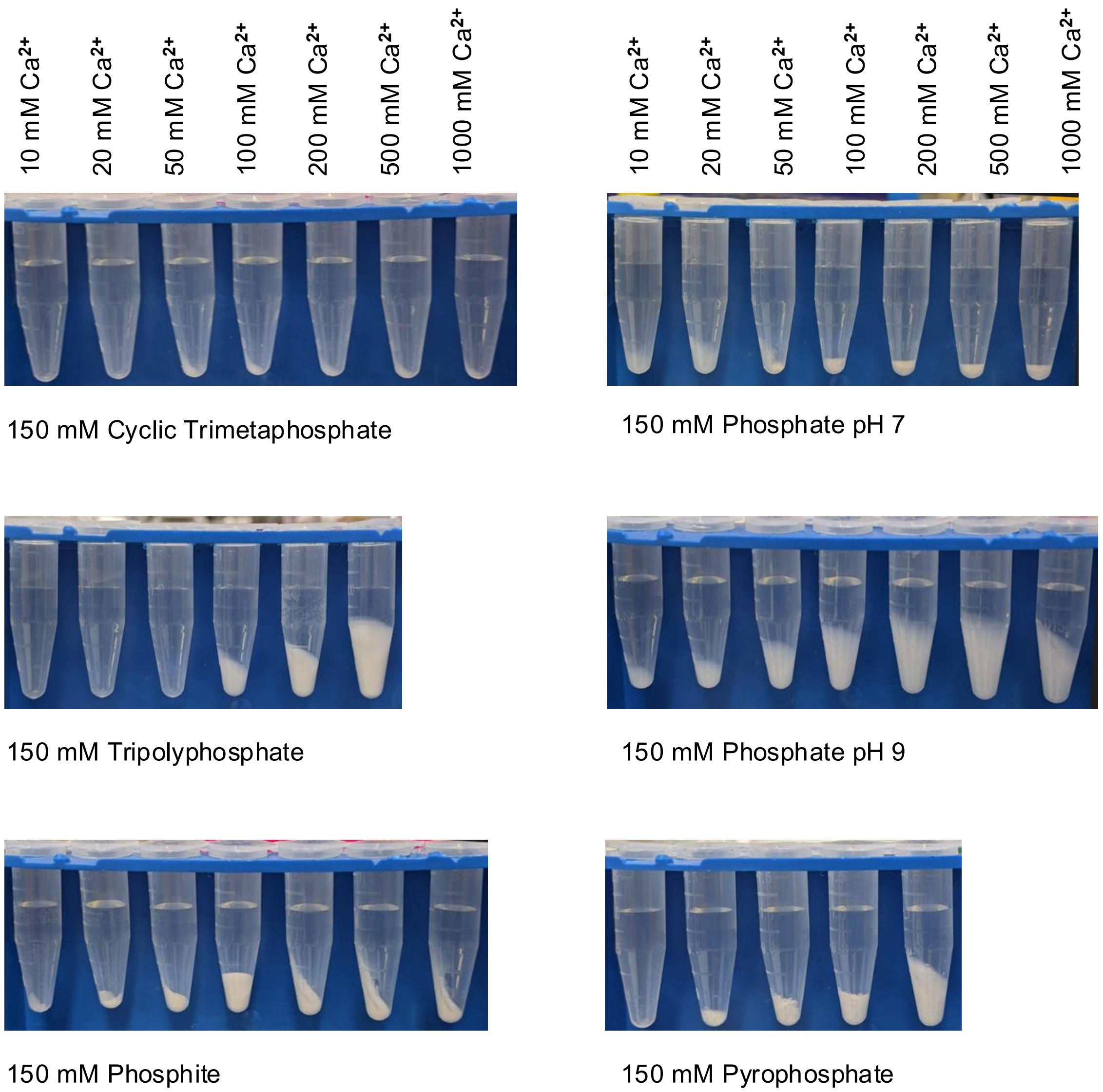

(F.) 1 mL total reaction volumes containing 150 mM of each phosphate species and varied Ca^{2+} concentrations, incubated overnight and centrifuged at 11000 g for 5 minutes. Due to solubility and volume constraints 1000 mM Ca^{2+} was not included in the case of tripolyphosphate, and 500 and 1000 mM Ca^{2+} were not included in the case of pyrophosphate.

Figure S5

G.

10 mM Ca^{2+} | 20 mM Ca^{2+} | 50 mM Ca^{2+} | 100 mM Ca^{2+} | 200 mM Ca^{2+} | 500 mM Ca^{2+} | 1000 mM Ca^{2+}

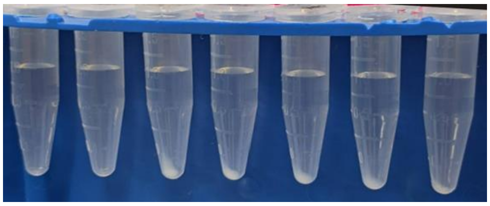

200 mM Cyclic Trimetaphosphate

10 mM Ca^{2+} | 20 mM Ca^{2+} | 50 mM Ca^{2+} | 100 mM Ca^{2+} | 200 mM Ca^{2+} | 500 mM Ca^{2+} | 1000 mM Ca^{2+}

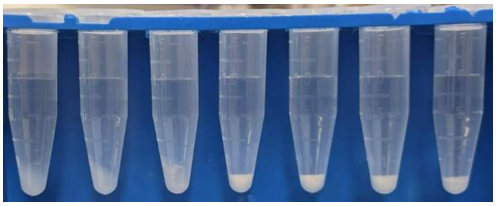

200 mM Phosphate pH 7

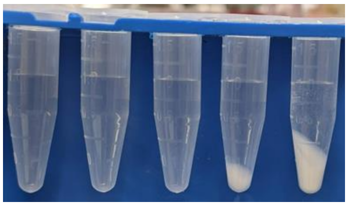

200 mM Tripolyphosphate

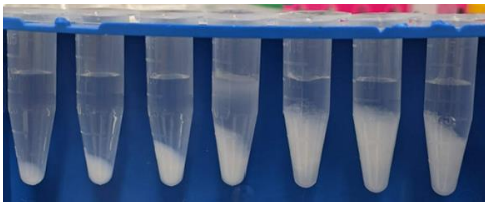

200 mM Phosphate pH 9

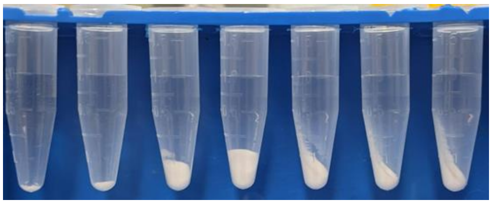

200 mM Phosphite

(G.) 1 mL total reaction volumes containing 200 mM of each phosphate species and varied Ca^{2+} concentrations, incubated overnight and centrifuged at 11000 g for 5 minutes. Due to solubility and volume constraints 500 and 1000 mM Ca^{2+} were not included in the case of tripolyphosphate. Pyrophosphate is not soluble in water at 200 mM and was not included.

Figure S6

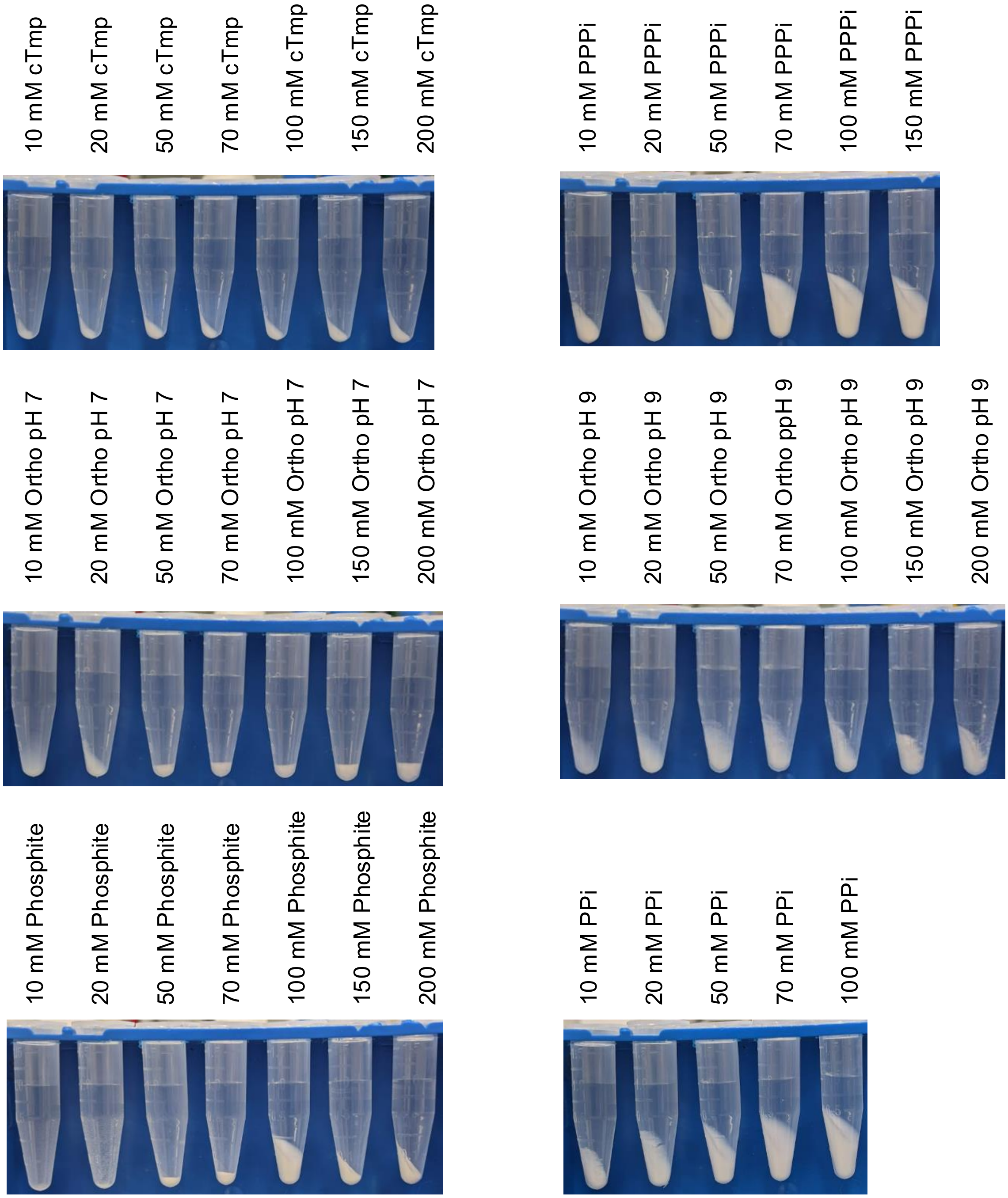

1 mL total reaction volumes containing prebiotic seawater solution and varied cyclic trimetaphostphate (cTmp), tripolyphosphate (PPPi), phosphate pH 7 (ortho pH 7), orthophosphate pH 9 (ortho pH 9), phosphite, and pyrophosphate (PPi) concentrations, incubated overnight and centrifuged at 11000 g for 5 minutes. Due to solubility and volume constraints 200 mM PPPi was not included, and 150 and 200 mM PPi were not included.
